# The Evolutionarily Conserved Serine Residues in BRI1 LRR Motifs Are Critical for Protein Secretion

**DOI:** 10.3389/fpls.2020.00032

**Published:** 2020-02-06

**Authors:** Tianshu Chen, Bin Wang, Fangfang Wang, Guanting Niu, Shuo Zhang, Jianming Li, Zhi Hong

**Affiliations:** ^1^ State Key Laboratory of Pharmaceutical Biotechnology, School of Life Sciences, Nanjing University, Nanjing, China; ^2^ Department of Molecular, Cellular, and Developmental Biology, University of Michigan, Ann Arbor, MI, United States; ^3^ College of Forestry and Landscape Architecture, South China Agricultural University, Guangzhou, China

**Keywords:** leucine-rich-repeat receptor-like kinases, BRI1, evolution, *Arabidopsis thaliana*, protein secretion, BR signaling

## Abstract

As a well-studied leucine-rich-repeat receptor-like kinases (LRR-RLKs) in Arabidopsis (*Arabidopsis thaliana*), BRI1 functions as a cell surface receptor for sensing the smallest ligand molecule identified thus far. The weak allele *bri1-9* (S662F) harbors a mutation at the conserved serine (Ser*) residue among 25 LRRs, which leads to the protein retention in the ER. However, very little is known about the importance of these residues. Through site-directed mutagenesis and a phenotypic complementation test, we examined the effects of these conserved serine residues (S*-chain) on protein secretion and functions. The results showed that the replacements of these serine residues significantly changed the sub-localization of BRI1-GFPs to the ER and that rigid space constraints, as well as the requirement of successive inner polar contacts, affect these sites. In addition, the continuous presence of Ser* is mainly disrupted at the LRR-island domain interface, and the changes of these four nonserine residues to serine greatly decreased the protein ability to complement *bri1-301* compact phenotype and the BR signaling activation. The sequence alignment revealed that other known LRR-RLK also harbors the S*-chain and the non-Ser* residues at the ligand-binding region along the S*-chain, which confirms the evolutionary significance of residues at these sites in plant LRR-RLKs.

## Introduction

As the largest family of cell membrane-localized receptor-like kinases (RLKs), leucine-rich-repeat (LRR) RLKs sense various internal and external signals to regulate multiple developmental processes and responses to environmental stresses (Shiu et al., 2004; Afzal et al., 2008; Lehti-Shiu et al., 2012; Smakowska-Luzan et al., 2018). LRR-RLKs are composed of an extracellular LRR domain responsible for ligand binding, a single membrane-spanning helix, and a cytoplasmic kinase domain (KD) (Afzal et al., 2008). In Arabidopsis (*Arabidopsis thaliana*), 200 members have been classified into 15 subgroups (SGs) based on a phylogenetic analysis of the KD sequences (Shiu et al., 2004; Lehti-Shiu et al., 2009; Wu et al., 2016). In flowering plants, SG_I, SG_VIII-2, SG_X, and SG_XI expanded considerably (Fischer et al., 2016; Liu et al., 2017), indicating that an extensive selection pressure is imposed on the LRR domain (Tang et al., 2010; Fischer et al., 2016). Plant LRR-RLKs share canonical plant-specific LRR consensus sequences (CS): LxxLxxLxLxxNxL(s/t)GxLPxxLGx (x represents any amino acid) (Kajava, 1998; Kobe and Kajava, 2001) and those harboring over 20 LRRs tend to form a plant-specific full turn superhelical assembly due to the formation of a second β-strand based on the L(s/t)GxLP motif (Kobe and Kajava, 2001; Shiu and Bleecker, 2003). Recently, the binding mechanisms of several LRR-RLK-ligand and LRR-RLK-ligand-coreceptor complexes were structurally investigated in Arabidopsis and the importance of the residue configuration of the LRRs on LRR-RLKs for proper function was shown. Ligands bind at the inner surfaces of LRR superhelical structures and recruit the coreceptors to activate signaling in a structure complementary way (Hohmann et al., 2017; Song et al., 2017; Hohmann et al., 2018).

Brassinosteroid insensitive 1 (BRI1) is a well-studied LRR-RLK that functions as a cell surface receptor for brassinosteroids (BRs) (Li and Chory, 1997; Kinoshita et al., 2005). Dysfunction of BRI1 or BR biosynthetic enzymes gives rise to a wide spectrum of growth defects, such as reduced seed germination, a dwarf stature, de-etiolation in the dark, delayed flowering, and male sterility (Clouse and Sasse, 1998; Vert et al., 2005; Fridman and Savaldi-Goldstein, 2013). The BRI1 extracellular domain consists of 25 LRRs, forming a superhelical assembly with a rise of 70Å and a 70-amino acid island domain inserted between the 21st and 22nd LRRs folds back into the interior of the superhelix and interacts extensively with LRRs 13–25 (Hothorn et al., 2011; She et al., 2011). The BR molecules bind to a hydrophobic groove between the island domain and the concave side of the BRI1 LRRs, together with the conformationally rearranged island domain, contributes to BRI1 heteromerization with BRI1-associated receptor kinase 1 (BAK1) (Nam and Li, 2002; Hothorn et al., 2011; She et al., 2011; Santiago et al., 2013; Hohmann et al., 2018). BR binding activates the signaling cascade and dephosphorylation of transcription factors BR-induced BRI1-EMS-suppressor1 (BES1) and brassinazole resistant 1 (BZR1) to regulate the BR-responsive gene expression (Wang et al., 2002; Yin et al., 2002). Several alleles with mutations either in the island domain or at the island-LRR interface have been identified, namely, *bri1-9* (Ser662Phe, S662F) (Noguchi et al., 1999), *bri1-113* (Gly611Glu, G611E) (Li and Chory, 1997), and *bri1-6* (Gly644Asp, G644D) (Noguchi et al., 1999). The structure analysis reveals that these mutations probably interfere with local conformations or hydrogen-bonding networks with BR diol moiety and consequently generate a negative effect on the recognition of BRs by BRI1 (Hothorn et al., 2011; She et al., 2011). Among these, bri1-9 is a structurally imperfect but functionally competent BR receptor that is recognized by endoplasmic reticulum (ER) resident lectins and chaperones, UDP-glucose: glycoprotein glucosyltransferase (UGGT) (Jin et al., 2007), calreticulin 3 (CRT3) (Jin et al., 2009), and BiPs (Jin et al., 2007; Hong et al., 2008). Unlike bri1-9, both bri1-6, and bri1-113 are localized at the plasma membrane (PM) (Hong et al., 2008). Bri1-9 harbors the mutation at Ser662 in the 22nd LRR, which is highly conserved among 25 LRRs of BRI1 and occupies the 10th position in the L^1^xxL^4^xxL^7^xL^9^S^10^xN^12^xL^14^(S/T) Gx^18^IPxx^22^LGx consensus motif (Li and Chory, 1997; Jin et al., 2007). However, little is known about the functions and the evolutionary significances of these highly conserved serine residues in BRI1.

In the current study, we investigated the roles on protein secretion and functions of the conserved serine residues lying along the inner concave surface of BRI1 LRRs. In addition, the nonserine residues (Gln424, Trp472, Asp496, and Asn568) disrupting the continuous serine contacts at the LRR-island domain interface were also studied. Our results strongly suggest that the conserved serine residues are crucial for maintaining BRI proper structure and the variation of these serine residues is likely to be correlated with BRI1 function.

## Materials and Methods

### Plant Materials and Growth Conditions

Arabidopsis ecotype Columbia (Col-0) and mutant plant *bri1-301* (Nam and Li, 2002) were used for *pBRI1:BRI1-GFP* and *pBRI1:bri1-GFPs* transformation. The seed surface sterilization and germination were conducted as previously described (Li et al., 2001). The seedlings were grown in culture room at 20℃ with 16-h light/8-h dark photoperiod.

### Construction of the BRI1 3D Model

Homology modeling of serine to other residue substitutions and other residues to serine substitutions (residues 37–770) was obtained *via* MODELLER program (https://salilab.org/modeller/) (Eswar et al., 2008) with BRI1 (PDB 3RGX She et al., 2011) as the template, followed the base modeling tutorial. The generated PDB files were visualized and labeled with PyMol (http://www.pymol.org/pymol).

### Plasmid Constructs and Plant Transformation

The *bri1-GFP* variants were generated from *pPZP212-BRI1:BRI1-GFP* (Friedrichsen et al., 2000) *via* site-directed mutagenesis using Quick Change II XL Site-Directed Mutagenesis kit (Stratagene, USA). The primers used for site-directed mutagenesis were listed in **Table S3** and the resulting plasmids were fully sequenced to ensure no additional PCR-introduced errors. The *bri1-GFP* variants were transformed into Arabidopsis wild-type Col-0 and *bri1-301* mutant (Nam and Li, 2002) *via* the *Agrobacterium tumefaciens*-mediated floral dipping method (Clough and Bent, 1998).

### Kif Treatment

For kifunensine (Kif) treatment, 2-week-old *bri1-301* seedling lines expressing similar level of *BRI1-GFP* and *bri1-GFPs* were removed from solid 1/2 Murashige and Skoog (MS) (Duchefa, Holland) medium, incubated in half-strength 1/2 MS (Duchefa, Holland) medium supplemented with or without 10 μM Kif (Sigma-Aldrich, USA) for continued growth, and removed 5 days later for photographing and protein extraction.

### Endoglycosidase H (Endo H) Treatment and Western Blot Analysis

Leaf tissues were ground in liquid nitrogen and extracted with 2×SDS sample buffer [0.125 M Tris (pH 6.8), 4% SDS, 20% glycerol, 0.2 M DTT, 0.02% (w/v) bromophenol blue]. The lysates were mixed and denatured at 97°C for 5 min. After centrifuged at 10000 × g for 10 min, the supernatant was treated with or without Endo H (New England Biolabs, USA) treatment for 1 h at 37°C, following the manufacture's procedure. Samples were then separated on 6.5% (BRI1-GFP) SDS-PAGE gel and transferred onto PVDF membrane (Roche Diagnostics, USA) for immune detection. Polycolonal antibody against GFP (Abclonal, China) was used to detect BRI1-GFP expression. Three independent replicates were conducted and representative western blotting images were shown.

### Root Inhibition Assay

The surface-sterilized seeds of T2 generation of *bri1-301* expressing different *bri1-GFP* from five representative T1 lines (ten seeds per line) were plated on the medium supplemented with (+) or without (−) eBL (Sigma-Aldrich, USA). After cold treatment for 2 days, the plates were transferred into growth chamber (22°C with a 16-h light/8-h dark photoperiod) and vertically grown for another five days. The seedlings were photographed, the primary root length was collected in Image J, and the relative root length to the control was analyzed. Data were analyzed using TTEST in Excel. More than 30 independent seedlings were used for the statistical analysis and three duplicate experiments were conducted.

### Sequence Collection, Alignment, and Phylogenetic Analysis

The Arabidopsis BRI1 protein sequence was used as a query to perform BLASTP search for homologous sequences in eight angiosperm genomes (*Amborella trichopoda* v1.0, *Oryza sativa* v7_JGI, *Brachypodium distachyon* v3.1, *Arabidopsis lyrata* v2.1, *Brassica rapa* v1.3, *Medicago truncatula* Mt4.0v1, *Solanum lycopersicum* iTAG2.4, and *Arabidopsis thaliana* TAIR10, Phytozome v12.1, https://phytozome.jgi.doe.gov), and also a gymnosperm genome (*Picea abies* v1.0, http://congenie.org/). The cDNA sequences of the top 20 hits in each angiosperm genome as well as 10 hits from *Picea abies* were identified and downloaded (**Table S4**). Under the guidance of kinase domain sequences, alignments were performed using ClustalW and MUSCLE programs in Mega 5.0 (Tamura et al., 2011), and the output was further optimized manually. The phylogenetic tree was constructed using the maximum likelihood (ML) method in Mega 5.0. A total of 100 bootstrap replications were performed to test the robustness of internal branches. Thirty-two pieces of sequences belonging to the BRI1 clade were then realigned with full coding sequences to build another phylogenetic tree, following the procedure mentioned above. The probability of conserved serines being present at the sites was also calculated from 32 sequences. To infer the direction and strength of natural selection on AtBRI1 and its close homologs, the ratio of nonsynonymous/synonymous substitution rate, denoted as dN/dS was calculated in Mega 5.0 with the modified Nei-Gojobori method (proportion) model.

## Results

### The Random Ser to Phe Substitutions Have Little Impact on BRI1 Subcellular Localization

To figure out whether the ER retention of bri1-9 arose from the substitution of a small hydrophilic serine (Ser, S), by a large, hydrophobic phenylalanine (Phe, F) residue, we examined the subcellular localization of three reported Ser to Phe mutations (S157F, S196F, and S399F) (Hong et al., 2008; Shang et al., 2011; **Table S1**), which all lied at variable sites of BRI1 LRRs. The S399F (bri1-120) has been shown to cause growth defects in Arabidopsis, suggesting that mutation has negative effect on protein function (Shang et al., 2011), whereas the function of the S196F and S157F have not been reported yet (Hong et al., 2008). From modeling, it was found that three sites lie at different regions of the BRI1 extracellular domain, to be specific, the concave side (Ser157), the convex outer surface (Ser196), and the surface buried under the island domain (Ser399) (**Figure S1A**, colored in magenta). The moiety exposures of Phe on surface were observed in S157F, S196F, and S662F (bri1-9) substitutions, but not in S399F, in which the exposure was covered by the island domain (**Figure S1A**, colored in magenta). We employed the Endoglycosidase H (Endo H) assay, a hydrolase to remove high-mannose and some hybrid Asn-linked glycans without fucose or xylose modification, to characterize the protein targeting (Shen et al., 2014). This is essentially a mobility shift assay based on the fact that the Asn-linked glycans on the ER-localized BRI1 alleles are sensitive to Endo H and will run faster after treatment with the enzyme, unlike the PM-localized BRI1, which are resistant to Endo H due to the maturation of the Asn-linked glycans in the Golgi that leads to complex glycans (Jin et al., 2007; Hong et al., 2009). The designated Ser to Phe mutation was individually introduced through site-directed mutagenesis method into an expression plasmid that has the *BRI1-GFP* gene under the regulation of *BRI1*'s native promoter (Friedrichsen et al., 2000). After transformation, leaves from five independent T1 seedlings were collected for total protein extraction and western blot analysis. We used Endo H-treated BRI-GFP and bri1-9-GFP as biomarkers to indicate the bands at PM and ER and focused on the band pattern of the proteins under the treatment with Endo H. Contrary to the modeling, we observed that all three BRI1-GFP variants carrying a Ser to Phe mutation showed the same band shift pattern as the wild-type BRI1-GFP (WT) (**Figure S1B**), indicating that these mutations had no impact on BRI1 conformation and BRI1 proteins secreted to PM normally. Therefore, the exposure of a bulky aromatic Phe residue was not sufficient for BRI1 mistargeting and the Ser662 site has a special contribution to BRI1 conformation.

### The Impact of Ser662 Residue on BRI1 Conformation Is Size-Dependent

**Figure 1:**
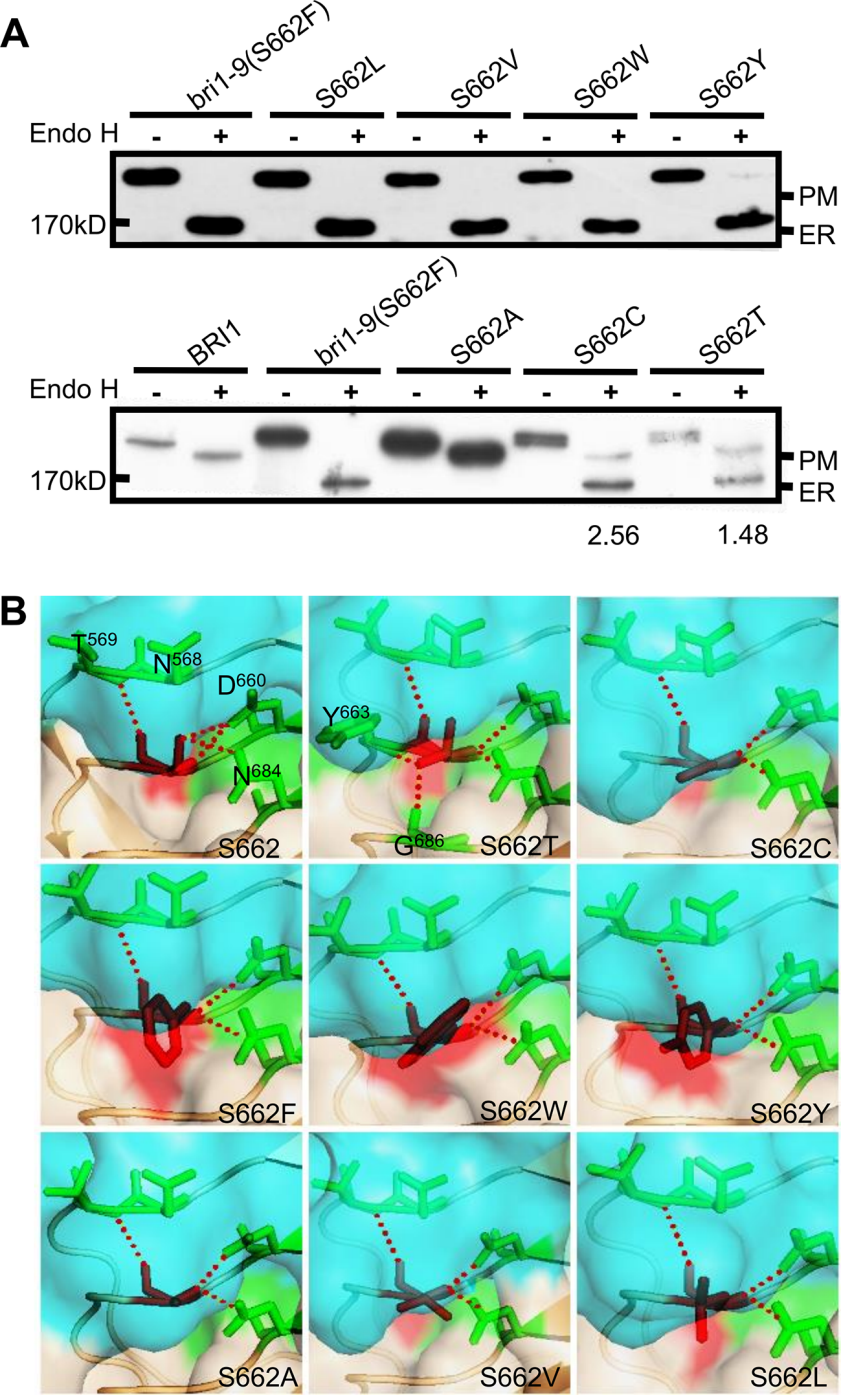
Subcellular localization of BRI1-GFP with substitution at Ser662. **(A)** Endo H assay to evaluate the subcellular localization of BRI1-GFP with replacement at Ser662. Total proteins from wild-type (WT) seedlings expressing BRI1-GFP and BRI1-GFPs were treated with (+) or without (−) Endo H to differentiate the mobility shift. The bands corresponding to BRI1-GFP at PM and ER were marked. The ratio of the endoplasmic reticulum (ER)/plasma membrane (PM) localized BRI1-GFPs was analyzed in ImageJ. **(B)** The structural models of substitution at Ser662. BRI1 (PDB 3RGX She et al., 2011) was used as the template, the simplified backbone was depicted in cartoon mode, and the solvent-accessible surface adjacent to Ser662 was shown in surface mode with 0.6 transparency. The replacements are denoted as red sticks and the poplar contacts between residues are displayed with red dotted lines.

To evaluate the importance of Ser662 in maintaining BRI1 conformation, we examined the specificity of this site with respect to the amino acid size, hydrophobicity, and the inner polar contact with surrounding amino acids. We first replaced the hydrophilic (polar) Ser residues with hydrophobic Leu (S662L), Val (S662V), aromatic residues Trp (S662W), and a large hydrophilic Tyr (S662Y). Western blot analysis showed that four kinds of substitutional BRI1-GFPs were all sensitive to Endo H treatment, similar to bri1-9 (S662F) (**Figure 1A**). As the crystal structure showed that Ser662 is located within a small pocket (She et al., 2011), we therefore speculated that this site is likely to be very strict in the size of amino acids. To test our hypothesis, we prepared the Ala substitution (S662A), which has a smaller side chain than that of Ser. As expected, the Endo H resistant pattern as WT was observed (**Figure 1A**), implying that S662A replacement had little effect on the BRI1 correct localization. In addition, we also individually changed the S662 to hydrophilic Cys (S662C) and Thr (S662T), which have slightly bigger side chains than Ser. Again, these two replacements obviously changed the BRI1-GFPs localization because two bands corresponding to ER and PM localized BRI1 forms were detected after Endo H treatment (**Figure 1A**), further confirming a rigid size requirement at this position. According to the structural models, S662 showed a polar contact with Asn568 (N568), Thr569 (T569), Asp660 (D660), and Asn684 (N684) (**Figure 1B**), and the introduction of mutations might destroy the polar contacts except for Thr662, which displayed extra polar contacts with the surrounding residues Tyr663 (Y663) and Gly686 (G686) (**Figure 1B**). The surface exposures of the residue at Ser662-site were predicted by the MODELLER program and were viewed in the solvent-accessible protein surface mode using PyMol (https://pymolwiki.org). According to the structural model, the surface exposure was S662W > S662Y ≈ S662F (bri1-9) > S662L > S662V > S662T > S662C ≈ S662 > S662A (**Figure 1B**, shaded in red), in agreement with the findings from the Endo H assay. Together, we concluded that an upper limit for space at the Ser662-site is important for BRI1 conformation.

### The Ser662-Localized Conserved Serine Residues Are Crucial for Stabilizing the Structure of BRI1

**Figure 2:**
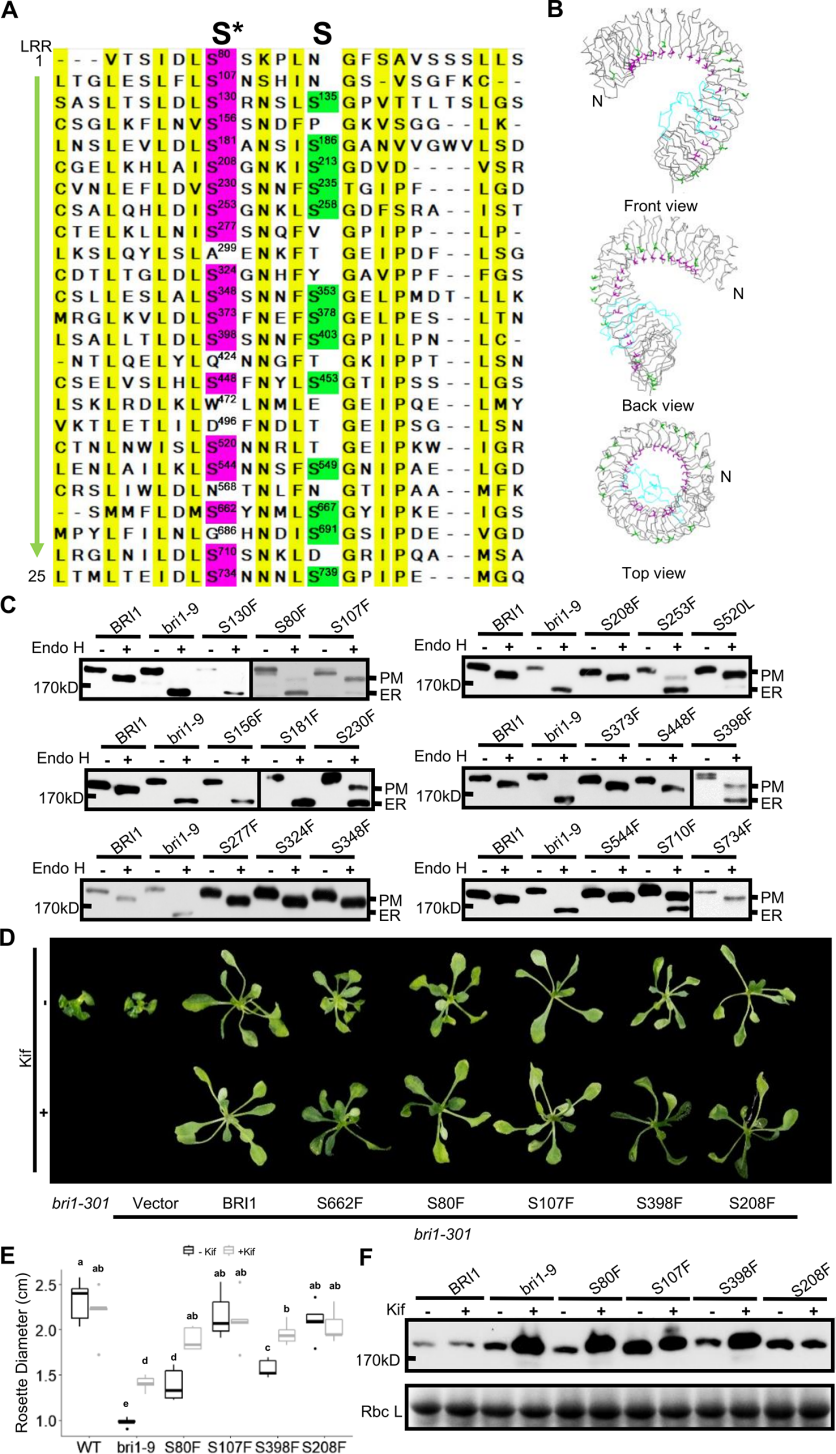
Single substitutions at conserved Ser residues greatly changed BRI1-GFP sublocalization to the endoplasmic reticulum (ER). **(A)** The sequence alignment of the BRI1 LRR domain. BRI1 extracellular domain consists of 25 LRR repeats. The two sets of conserved Ser residues lying in the concave inner side (S*-chain) and the convex outer side (S-chain) are highlighted in magenta and green, respectively. The consensus residues are highlighted in yellow. **(B)** The line-model views of S*-chain (in magenta) and S-chain residues (in green) on BRI1 extracellular structure. The simplified backbone was depicted in ribbon mode, the residues along the S*-chain and S-chain residues were shown in magenta and green line modes, respectively. The island domain is depicted in light blue. **(C)** The Endo H assay of BRI1-GFP variants carrying S to F mutation along S*-chain. The WT seedlings expressing *BRI1-GFP* and *bri1-GFPs* harboring single S to F substitution were treated with or (+) without (−) Endo H to detect protein mobility shifts. The bands representing BRI1-GFP on plasma membrane (PM) and in the ER were marked. **(D)** The BRI1-GFP variants were functional. Two-week-old *bri1-301* seedlings expressing similar levels of BRI1-GFPs grown on 1/2 MS medium were treated with (lower) or without (upper) 10 μM Kif for five days and representative phenotypes were shown. Three independent replicates were conducted and representative seedlings were shown. **(E)** The statistical analysis of the rosette diameters. The seedlings treated as in **(D)** were collected and the diameters of the rosettes (n > 10) were collected in Image J. Data were analyzed using TTEST in Excel. The letters indicated statistical differences (P < 0.05) (n > 10). **(F)** The accumulation of BRI1-GFPs against Kif treatment. The seedlings shown in **(D)** were harvested for protein extraction and gel blot.

It is known that Ser662 lies at a highly conserved site among 25 LRRs of BRI1 (Li and Chory, 1997; Jin et al., 2007). The alignments of BRI1 LRRs showed that 19 out of a total of 25 LRRs are occupied by Ser residues, including S662 along the inner side of superhelix and forming a continuous Ser chain (**Figures 2A, B**, colored in magenta), named as S*-chain in the current study. We found that the Ser residues along the S*-chain (denoted as Ser*) also formed a hydrogen bond network together with adjacent Asp (D) residues to stabilize the assembly of repeating LRR motifs (**Figure S2**). To decipher if Ser* residues had a similar impact on BRI1 structural maintenance, we individually mutated these Ser* to Phe, analogous to bri1-9 (S662F). We failed to generate S520F but did S520L instead. The results showed that 10 out of 18 new substitutions affected the normal PM-localization of BRI1-GFP, which was reflected in the presence of an Endo H sensitive band (ER-localized BRI1-GFP). Notably, Ser residues lying in the N terminal LRRs seemed to be the most important to maintain BRI1 conformation since substitutions from LRR1 to LRR8 led to obvious ER retention of BRI1-GFPs except S208F (**Figure 2C**, **Table 1**). From western blot analysis, mistargeting of BRI1-GFPs showed distinct levels of ER retention, i.e., the majority of the proteins, half or less than half of the proteins were found to be in the ER. The sublocalizations of some BRI1-GFPs (S80F, S107F, S208F, and S398F) were further confirmed by confocal microscopy images of Arabidopsis seedlings stably expressing *pBRI1:bri1-GFPs* and *Agrobacterium*-infiltrated tobacco leaves (**Figure S3A, B**). An obvious colocalization of BRI1-GFPs with the ER marker HDEL-RFP was observed (**Figure S3B**).

**Table 1:** Summary of highly conserved Ser* residues replaced by Phe and Thr on BRI1-GFPs and the corresponding effects on sub-localization.

Mutation	LRR	Subcellular localization	Change of subcellular localization for F-T mutation
**S80F**	**1**	**ER**/PM = 2.35 ± 0.85	**PM**
**S107F**	**2**	ER**/PM =** 0.53 ± 0.09	ER**/PM =** 0.12 ± 0.05
**S130F**	**3**	**ER**	**ER/**PM = 2.55 ± 0.95
**S156F**	**4**	**ER**	ER**/PM =** 0.62 ± 0.09
**S181F**	**5**	**ER**	**PM**
**S208F**	**6**	**PM**	
**S230F**	**7**	**ER/**PM = 1.51 ± 0.50	ER**/PM =** 0.23 ± 0.05
**S253F**	**8**	**ER/**PM = 3.78 ± 0.75	**PM**
**S277F**	**9**	**PM**	
**S324F**	**11**	**PM**	
**S348F**	**12**	**PM**	
**S373F**	**13**	**PM**	
**S398F**	**14**	**ER/**PM = 1.53 ± 0.47	**PM**
**S448F**	**16**	**PM**	
**S520L**	**19**	ER**/PM** = 0.09 ± 0.05	
**S544F**	**20**	**PM**	
**S662F**	**22**	**ER**	
**S710F**	**24**	ER**/PM** = 0.56 ± 0.18	**PM**
**S734F**	**25**	**PM**	

Bold characters indicate increased subcellular localization. The ratio of the gray value of the ER/PM localized bands were analyzed in ImageJ in at least three western blotting experiments.

We also did a complementation test to examine the effect of Ser* on the BRI1 function. The BRI1-GFPs harboring S80F, S107F, S208F, and S398F were individually introduced into a weak allele, *bri1-301* (Nam and Li, 2002; Zhang et al., 2018). Although all of the tested BRI1-GFP variants could suppress the compact rosette leaf phenotype of *bri1-301*, the degree of phenotypic recovery by BRI1-GFPs harboring S80F or S398F mutations was quite similar to that of S662F and the complementation achieved by BRI1-GFP carrying S107F or S208F mutations was comparable to that of wild type BRI1 (**Figures 2D, E**). This result was in good accordance with the western blot analysis in which BRI1-GFPs harboring S107F or S208F showed more PM-localization, whereas BRI1-GFPs carrying S80F and S398F were mainly localized in the ER, like S662F (bri1-9) (**Figure 2C**). We then treated the seedlings with kifunensine (Kif), the inhibitor of ER-mannosidase I, to prevent ER-associated degradation. Five days after Kif treatment, the seedlings of S662F (*bri1-9*), S80F, and S398F exhibited a more expanded rosette leaf phenotype compared to the untreated group (**Figures 2D, E**). Correspondingly, an obvious accumulation of protein was observed in the seedlings transformed with BRI1-GFPs carrying S80F and S398F (**Figure 2F**), further implying that these BRI1-GFP variants are indeed located in the ER and are regulated by the ER-associated degradation (ERAD) machinery, like bri1-9, and are structurally imperfect but functionally competent BR receptors (Jin et al., 2007). These results revealed that the presence of the conserved S*-chain mainly contributed to BRI1 conformation instead of function. In addition, the possible relationship between the local LRRs hydrophobicity and the secretory pattern of the corresponding BRI1-GFP variants carrying a Ser* to Phe substitution was also investigated and no obvious correlation was found (**Table S2**).

### Size and Polarity Constraints Explain the Conservation of Ser* Residues at the Inner Side of BRI1 LRRs

**Figure 3:**
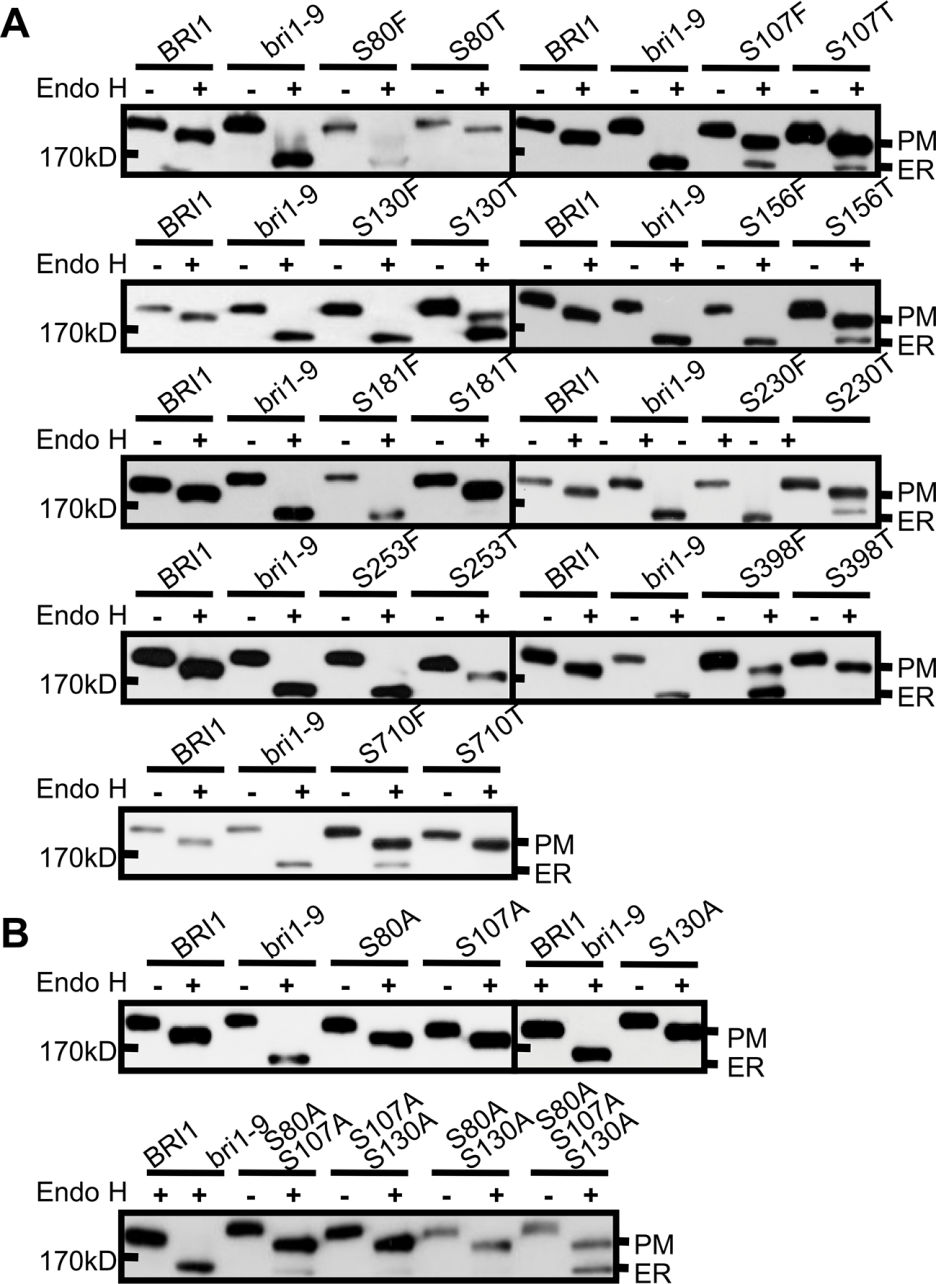
The rigid space constraints and polar contacts of the Ser* residues. **(A)** Endo H assay to evaluate the space constraint on Ser* residues. The subcellular localization of BRI1-GFPs harboring Ser to Thr replacements was detected and the replacements of Ser to Phe were used as controls. **(B)** The continuous substitutions of Ser* residues with Ala led to the endoplasmic reticulum (ER) retention of BRI1-GFP. WT seedlings expressing *BRI1-GFP* and *bri1-GFPs* were treated with (+) or without (−) Endo H and gel run for detecting protein mobility shift. BRI1-GFP and bri1-9-GFP were used as plasma membrane (PM)- and ER-localized indicator proteins, respectively.

To investigate the mechanisms underlying the conservation of Ser* residues in the LRRs, we first mutated to Thr (T) those Ser* residues that had been shown to change BRI1-GFP normal subcellular localization when changed to Phe. Compared with the Ser* to Phe substitutions, we found that the changes of Ser* to Thr decreased the BRI1-GFP sensitivity to Endo H, albeit four (S107T, S130T, S156T, and S230T) from nine mutated BRI1-GFP were still partially retained in the ER (**Figure 3A**, **Table 1**), supporting the previous finding that the size along the S*-chain is an important factor to keep BRI1 conformation. In addition, we checked the impact of successive polar contact/polarity of Ser* residues on BRI1 conformation or secretion. The replacements of Ser* to Ala at Ser80, Ser107, and Ser130 were individually generated (S80A, S107A, and S130A), and the Endo H assay showed that these BRI1-GFP variants were resistant to Endo H treatment, indicating a normal secretion to PM (**Figure 3B**). However, when these three Ser* residues were gradually mutated to generate a tandem of double or triple Ala mutations, a tendency for proteins to be trapped in the ER was detected and triple mutations had greatly changed the subcellular localization of BRI1-GFP from PM to the ER, as revealed by the presence of Endo H sensitive bands (**Figure 3B**), suggesting that the break of polar contacts impaired BRI1 conformation. Our results suggest that the conservation of Ser* residues along the chain is critical for the BRI1 structural maintenance and that the S*-chain fulfills the size and polarity requirements.

### The Variations on the S*-Chain Sites Reflect a Derived State Due to Selection

**Figure 4:**
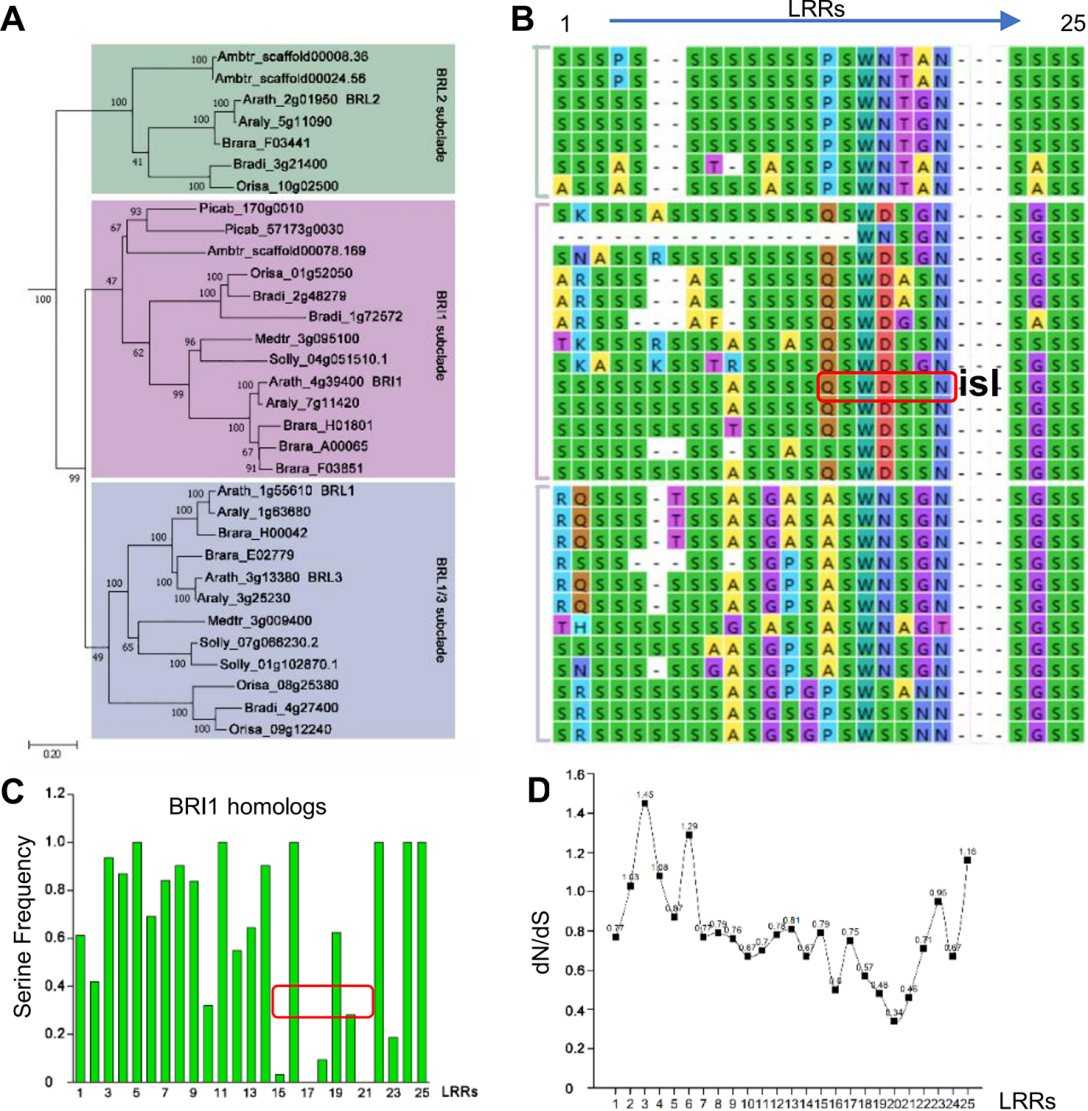
Phylogenic analysis of Ser* residues in BRI1. **(A)** The phylogenic analysis of BRI1 homologs from nine representative seed plants, including *Amborella trichopoda1* (Ambtr), *Oryza sativa* (Orysa), *Brachypodium distachyon* (Bradi), *Arabidopsis lyrate* (Araly), *Brassica rapa* (Brara), *Medicago truncatula* (Medtr), *Solanum lycopersicum* (Solly), *Arabidopsis thaliana* (Arath), and *Picea abies* (Picab). The bar indicated a mutation rate of 0.20 substitutions per site. Bootstrap values were shown near the nodes. **(B)** The Ser* residues among BRI1 LRR repeats and its homologs are listed in **(A)**. “isl” is the abbreviation of “island domain” and those residues marked in red box were reported to be involved in the formation of LRR-island domain interface. **(C)** The quantification of Ser* residues presenting on BRI1 and its homologs. The values were calculated with the Ser* appearance in each leucine-rich-repeat (LRR) and the area marked with red box corresponds to the LRRs shown in **(B)**. **(D)** dN/dS of each LRRs in AtBRI1 and AtBRL1-3. The ratio of nonsynonymous substitution rate to synonymous substitution rate, denoted as dN/dS or ω, was calculated in Mega to infer the direction and strength of natural selection.

Since the existence of the S*-chain seemed to be a necessity for Arabidopsis BRI1, here a question emerged: How were these Ser* residues selected during evolution? It is predicted that BRI1 might arise before the emergence of angiosperms and after the split between gymnosperms and the angiosperms (Wang et al., 2015). Therefore, to address this question, we searched BRI1 homologous sequences using the AtBRI1 protein sequence as a query and downloaded 170 cDNA sequences from the top hits in nine representative seed plants, including top 20 hits from each of the eight angiosperms (*Amborella trichopoda*, *Oryza sativa*, *Brachypodium distachyon*, *Arabidopsis lyrata*, *Brassica rapa*, *Medicago truncatula*, *Solanum lycopersicum*, and *Arabidopsis thaliana*) and all 10 hits form the gymnosperm genome *Picea abies* (**Table S4**). Phylogenetic analyses using the kinase domain of these sequences recovered two clear LRR-RLK clades (SG_X and SG_XI), as reported previously (Shiu et al., 2004). We then further selected 80 sequences belonging to the SG_X clade (containing the BRI1 family) to reconstruct a maximum likelihood (ML) tree using full coding sequences. As shown in **Figure 4A**, the BRI1/BRI1-like clade consisting of 32 sequences, could be separated into three subclades, namely, BRI1, BRL2, and BRL1/BRL3 subclades. In the BRI1 subclade, the sequences from *Picea abies* (Picab) exhibited a similar Ser* occupancy along the S*-chain as in AtBRI1 (**Figures 4A–C**), indicating that Ser* at these sites have been conservatively maintained in both gymnosperms and angiosperms.

In BRI1 LRRs, the analyzed positions are variable, and Ser* conservation was observed to be diminished from LRR15 to LRR21, in which the continual S*-chain was disrupted by Gln424 (Q424), Trp472 (W472), Asp496 (D496), and Asn568 (N568) at the corresponding sites (**Figures 4B, C**, in red boxes). From the dN/dS ratio (ω, the nonsynonymous/synonymous substitution rates) of individual LRRs between AtBRI1 and its three close homologs (**Figure 4D**), we found that the *ω* value from LRR15 to LRR22 was clearly lower than those of other LRRs, indicating that a stronger functional constraint (purifying selection) might have on these LRRs. Intriguingly, the nonserine residues Gln424, Trp472, Asp496, and Asn568 appeared along the S*-chain in these constrained LRRs. In contrast, in LRRs with higher *ω* value Ser residues appeared at corresponding sites.

### The Variable Residues Along the S*-Chain Are Involved in BR Signaling

**Figure 5:**
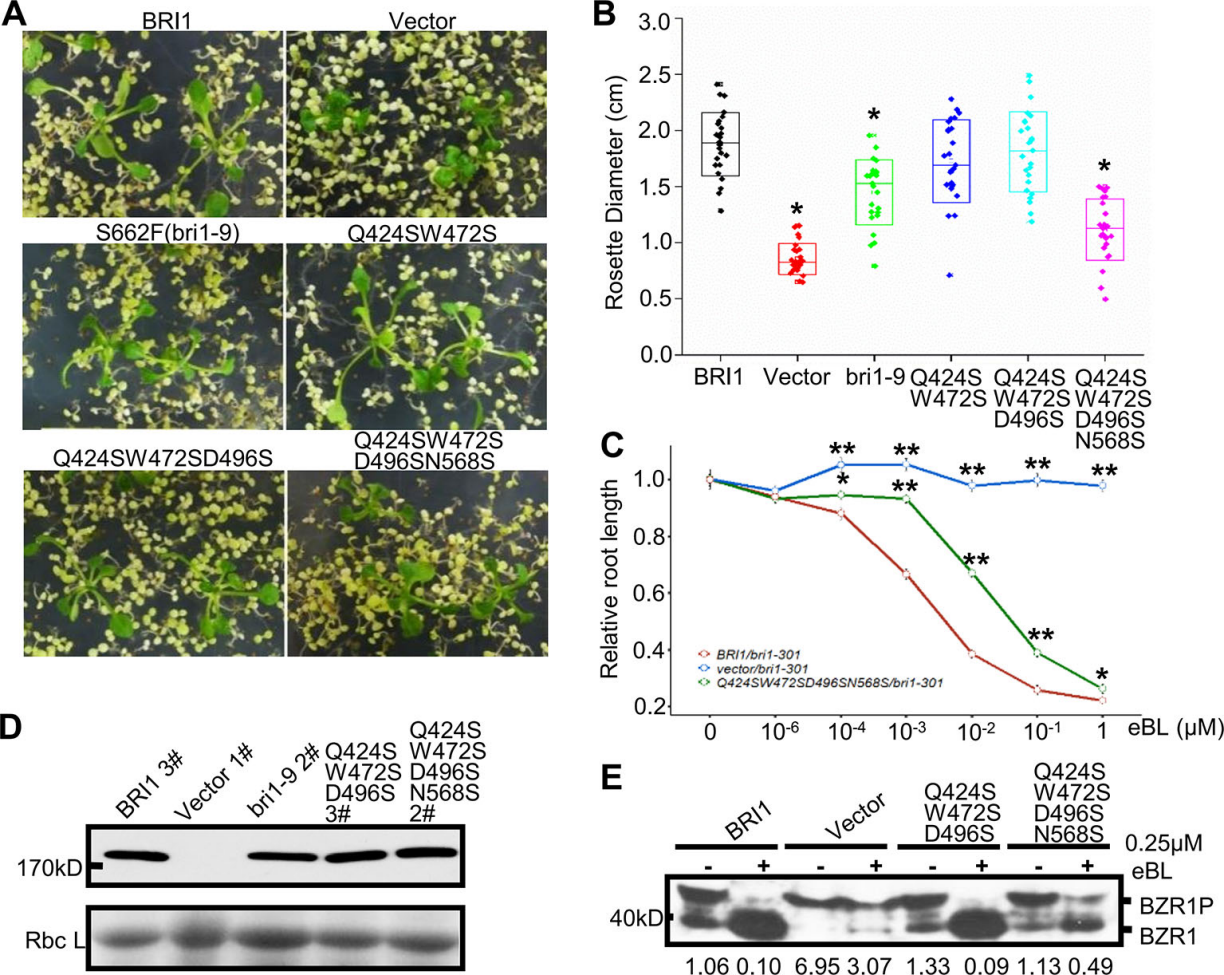
Functional analysis of the variable residues along the S*-chain. **(A)** Phenotype of *bri1-301* expressing *bri1-GFPs* harboring mutations on the variable residues along the S*-chain. Two representative T1 seedlings grown on selection medium for 3 weeks are shown under the same magnification. **(B)** The statistical analysis of the rosette diameter. T1 seedlings expressing *BRI1-GFP* or *bri1-GFPs* were screened and photographed after three weeks on selection medium and the diameters of the rosettes were collected in Image J. Data were analyzed using TTEST in Excel. More than 25 independent lines were used for the assay. Asterisk indicates statistical differences between *pBRI1:BRI1-GFPs* and *pBRI1:bri1-GFPs* lines (P < 0.05) (n = 25). **(C)** Root inhibition assay of *bri1-301* expressing *bri1-GFP* with quadruple mutations. Each data point denoted the average root length of seedlings. Error bar meant SE of three independent replicates. Data were analyzed using TTEST in Excel. Asterisks indicated statistical differences of the vector lines and the quadruple mutation lines to *pBRI1:BRI1-GFPs* lines under the same dose of eBL treatment (n = 30). “*” and “**” meant P < 0.05 and P < 0.01, respectively. **(D)** Western blot to detect BRI1-GFP expression. Total proteins were extracted from *bri1-301* seedlings expressing *BRI1-GFP* and *bri1-GFPs*, and detected with an anti-GFP antibody. **(E)** The dephosphorylation of BZR1. Two-week-old *bri1-301* seedlings expressing a similar level of various BRI1-GFPs in **(D)** were incubated in liquid 1/2 MS medium supplemented with (+) or without (−) 0.25 μM eBL (Sigma-Aldrich, USA) for 1 h. Each treatment included five seedlings and total proteins were separated on 10% SDS-PAGE gel for western blot using anti-BRZ1 antibody. The ratio between the phosphorylated and dephosphorylated BZR1 was analyzed with ImageJ.

To verify the possible correlation between these variations and the BRI1 function, we mutated Gln424, Trp472, Asp496, and Asn568 to serine residues and introduced these mutants into the *bri1-301* mutant line to observe the complementation phenotype. We found that the simultaneous substitution of four residues for serine resulted in an obvious decrease in the ability of the protein to rescue the *bri1-301* mutant phenotype, which showed compact rosette leaves (**Figures 5A, B**), suggesting that these four sites contribute to BRI1 function.

The Endo H assay revealed that the replacements had little effects on BRI1-GFP localization, even when all four sites were mutated simultaneously (**Figures S4A, B**), which suggests that the nonserine resides play a role in BRI1 function rather than acting as structural elements. Further evidence came from the root inhibition assay that the *bri1-301* seedlings transformed with BRI1-GFP carrying quadruple mutations showed less sensitivity to epi-brassinolide (eBL) compared to those expressing BRI1-GFP (**Figure 5C**). Moreover, when treated with 0.25 μM eBL treatment for 1 h, the dephosphorylation of BZR1 of the quadruple mutations is delayed compared to those expressing similar level of BRI1-GFP (**Figures 5D, E**). In contrast, complete dephosphorylation of BZR1 can be observed upon the treatment with 1 μM eBL for 1 h (**Figure S4C**).

### The Residues on the Less Conserved S-Chain Have Different Roles in BRI1 Structure Stability

**Figure 6:**
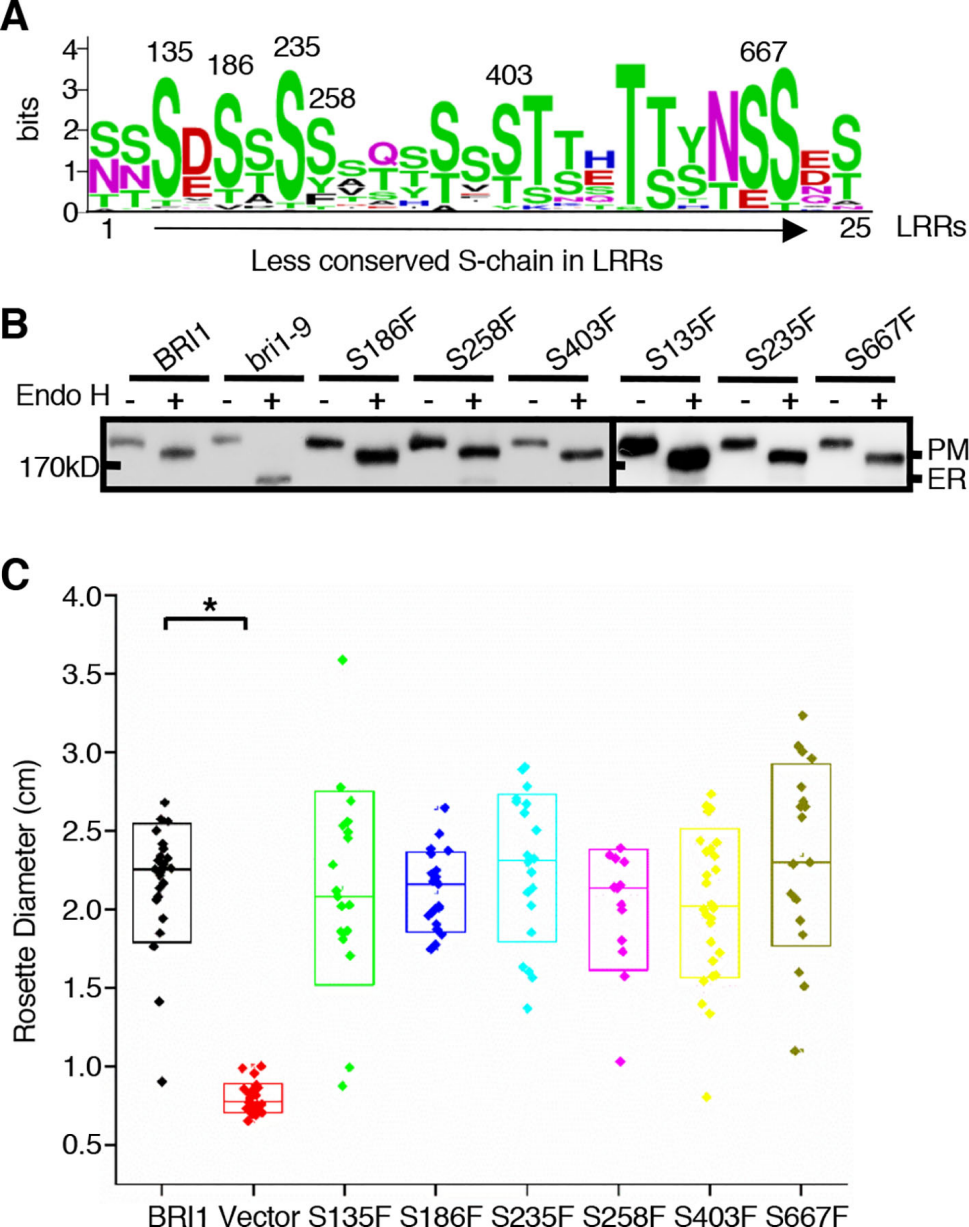
The effects of substitutions of less conserved Ser residues on the sub-localization of BRI1-GFPs. **(A)** Residue distribution along the less conserved Ser chain (S-chain). Thirty-two pieces of protein sequences from BRI1 homologs in **Figure 4A** were used to calculate the frequency of residue at each site in distinct leucine-rich-repeat (LRR) and displayed with WebLogo (http://weblogo.berkeley.edu/logo.cgi). The height of each symbol indicated the conservation (measured in bits). **(B)** Endo H treatment to detect the impact of less conserved Ser residues on BRI1 secretion. Total proteins from wild-type (WT) seedlings expressing BRI1-GFP and those carrying S to F mutant along the less conserved S-chain were treated with (+) or without (−) Endo H, and detected with an anti-GFP antibody. The bands corresponding to plasma membrane (PM)- and endoplasmic reticulum (ER)-localized counterparts of BRI1-GFPs were marked. **(C)** Phenotypic analysis of 3-week-old transgenic *bri1-301* seedlings expressing *pBRI1:BRI1-GFPs* and *pBRI1:bri1-GFPs*. The rosette diameters of 18~25 T1 transgenic lines were examined and shown in box plot. The asterisk indicates a statistically significant difference between *pBRI1:BRI1-GFPs* and *pBRI1:bri1-GFPs* lines (P < 0.01) (n = 18~25).

Notably, there were alternate conserved Ser residues in BRI1 LRR domain lying in the plant-specific L(S/T)GxLP motif to form the second β sheet at the convex surface of the super-helix (**Figure 2B**, colored in green). In contrast to the S*-chain, only 13 out of a total of 25 LRRs were occupied by Ser residues at the corresponding sites and formed a relaxed S-chain (**Figure 2A**, colored in green). Although these Ser residues showed diverse conservation among BRI1 and its homologs (**Figure 6A**), all six substitutions (S135F, S186F, S235F, S258F, S403F, and S667F) had little effect on the subcellular localization of BRI1 mutants fused to GFP since they all showed resistance to Endo H treatment similar to the WT, in contrast with those replacements in the corresponding S*-chain (**Figures 2C** and **6B**). To determine the function of these BRI1 alleles, the complementation test of *bri1-301* were carried out. The rosette diameters of 18~25 T1 seedlings were analyzed and no significant differences could be detected (**Figure 6C**), indicating that the Ser residues lying on the convex surface play different roles on BRI1 conformation and function.

## Discussion

In the current study, we investigated the function of a series of highly conserved Ser* residues along the inner surface of the BRI1 LRR solenoid. Our results showed that these Ser* residues are critical for maintaining the conformation of BRI1-LRRs (**Figure 2C**, **Table 1**) and both rigid spatial constraints and continuous inner polar contacts exist at these sites (**Figures 1** and **3**, **Figure S2**). Replacements on these sites are expected to perturb the local conformation and give rise to the exposure of internal hydrophobic residues. Notably, the Ser* residues at N-terminal LRRs (LRR1-LRR8) are more important because the replacements of Ser* to Phe or Thr significantly change the sub-localization of BRI1-GFPs to the ER (**Figures 2C** and **3**). By contrast, most serine residues lying in the middle are covered by island domain (**Figure S1A**), leading to the inaccessibility of the ER residential chaperones recognition. However, the continuous S*-chain is disrupted from LRR15 to LRR21, and the changes of four nonserines (Q424, W472, D496, and N568) back to serine had little impact on BRI1-GFPs PM localization but greatly decreased the protein ability to complement the *bri1-301* compact phenotype, indicating that the variation of the Ser* residues is closely correlated with BR signaling activation (**Figure 5**, **Figure S4**). The less conserved Ser residues are present along the convex outer surface of the BRI1 LRR domain, which forces the BRI1 LRRs to stack into a superhelical assembly (Kajava, 1998; Kobe and Kajava, 2001). Yet only weak polar interactions and relaxed spatial constraints could be observed among these dispersed Ser residues (**Figure 2B**, colored in green). Replacing these Ser residues with Phe has little impacts on the BRI1 structure or BR signaling pathway, in sharp contrast to the replacement of Ser* residues in same LRR repeat (**Figures 2** and **6**).

Intriguingly, contrary to the expected importance of this S*-chain, only three weak mutations [*bri1-9*, *bri1-706* (S253F) and *bri1-235* (S156F)] out of 19 Ser* sites have been genetically identified (Noguchi et al., 1999; Sun et al., 2017; Li et al., 2019). This inconsistency may be due to the fact that these Ser* residues serve as structural frameworks, and mutations on these sites do not affect BRI1 function. In a previous study, the *bri1-9* shows a dwarf phenotype and was thought to be a functional incompetent *BRI1* allele before it was reported to be recognized by ER resident chaperones and trapped in the ER (Jin et al., 2007). In the study, the authors use Endo H, confocal and two-phase partitioning assays to confirm the ER retention of bri1-9. However, the overexpression of bri1-9 or the inhibition of the ER-associated degradation pathway can rescue *bri1-9* mutant phenotype due to the leak of some bri1-9 from saturated ERQC, suggesting that bri1-9 is a functional receptor (Jin et al., 2007; Hong et al., 2009; Jin et al., 2009; Hong et al., 2012). These findings prove that the overvigilant ERQC system might trap and degrade the functional proteins with structural defects in the ER, leading to mutant phenotype. In the current study, all of the tested BRI1-GFP variants with distinct secretary pattern could complement *bri1-301* compact phenotype although some variants such as S80F and S398F, seem less functional than the wild-type BRI1 (**Figures 2D, E**). Therefore, mutations on these Ser* sites may induce indiscernible growth defects. The second possibility is that the presence of N-glycans on the protein surface may supply steric protections (Shental-Bechor and Levy, 2008). BRI1 is decorated with carbohydrates and a total of ten N-glycosylation sites have been identified in the extracellular domain. From structure, most Ser* residues are usually masked by N-glycans nearby especially for those located at the N termini, compared with the exposed S662 (Hothorn et al., 2011; She et al., 2011). It is quite possible that the N-glycans serve as steric clash to prevent the minor structurally imperfect assembly from being recognized by the ER-resident chaperones, therefore, BRI1 variants harboring a substitution at Ser* sites can normally or partially be secreted to the cell membrane for BR perception and only those mutations leading to a complete ER retention, such as S130F, S156F, and S181F (**Figure 2C**; **Table 1**), are expected to produce an easily identified dwarf phenotype similar to *bri1-9* (Jin et al., 2007).

Extracellular LRR domains are responsible for ligand binding and recruit a ligand-dependent co-receptor to trigger signal transduction (Hohmann et al., 2018). According to crystal structures, the ligands extend along the inner surface consisting of residues from the 4th to 12th sites (L^4^xxL^7^xL^9^xxN^12^) in LRR repeats (Hohmann et al., 2017), which has been indicated in some studies that these sites are positively selected (Wang et al., 1998; Zhang et al., 2006), especially the variable residues at the 6th, 8th, 10th, and 11th positions (Fischer et al., 2016). In the current study, we show that BRI1 has an evolutionarily conserved pattern at the 10^th^ site in 9 represented plants (**Figures 4A, B**). The highly conserved Ser* arrangement is disrupted by several nonserine residues mainly lying in LRR15 to LRR21 (**Figures 4B, C**). From the evolutional analysis, LRRs containing non-Ser are under a stronger functional restriction in comparison with those that have Ser* residues (**Figure 4D**). Four nonserine residues (Q424, W472, D496, and N568) in Arabidopsis BRI1 are proved to be closely correlated with the BRI1 function (**Figure 5**). Since these four nonserine residues are located in the LRR-island domain interface, two possible mechanisms may explain the involvement of these residues in BRI1 function. Upon ligand binding, the structure rearrangement occurs in the island domain to form a groove with the associated LRRs for BR binding (Hothorn et al., 2011; She et al., 2011) and the changes of Q424, W472, D496, and N568 may affect island domain reformation, therefore decreasing ligand binding efficiency. Alternatively, the recruitment of the coreceptor is based on extensive interactions with the island domain, BR molecules, and LRRs (Santiago et al., 2013; Hohmann et al., 2018). It is possible that four nonserine residues are involved in this process and the mutations may block the formation of this recruitment platform.

From sequence alignment, we found that the S*-chain also exists in other known LRR-RLKs with or without island domain and that non-Ser* residues at the ligand-binding region along the S*-chain are also present (**Figure S5A**, highlighted in red boxes). Similar to BRI1 LRRs, Ser* residues mainly appear at the N or C termini (**Figure S5B**). From crystal structures, Ser* residues form extensive polar interactions with adjacent aspartic acid (Asp, D) residues at the −2 sites to stabilize BRI1 LRRs (**Figures 2A, B**; **Figure S2**). Likewise, Asp residues tend to appear coincidentally with Ser* residues in other LRR-RLKs and the ratio of Asp-Leu-Ser* (D-L-S*) versus X-Leu-Ser* (X≠Asp, X-L-S*) ranges from 44% to 75% (**Figure S5A**). Recently, the D-L-S* motifs have also been reported in some LRR protein designs (Parmeggiani et al., 2015). Therefore, we presume that the serine residues of the S*-chain in LRR-RLKs might work as a structural frame and the nonserine residues might be involved in ligand binding or coreceptor recruitment. A better functional identification of these conserved residues on other LRR-RLKs may supply more clues to understand the relationship between protein structure and ligand recognition. This might also increase the potential for optimizing artificial modular binding in biomedical applications.

## Data Availability Statement

All datasets generated for this study are included in the article/**Supplementary Material**.

## Author Contributions

TC performed the experiments and wrote the draft. BW analyzed the phylogenic tree and revised the draft concerning the evolution. GN assisted in site-directed mutagenesis. JL, FW, and SZ help analyzing the data. ZH designed the project and revised the manuscript. The authors declare no competing financial interests.

## Funding

This work was supported by grants from the National Natural Science Foundation of China (No.31322008 and No.31870263). The authors declare no conflict of interest.

## Conflict of Interest

The authors declare that the research was conducted in the absence of any commercial or financial relationships that could be construed as a potential conflict of interest.
