# Supplementary material for: The Evolutionarily Conserved Serine Residues in BRI1 LRR Motifs Are Critical for Protein Secretion
Source: Front Plant Sci. 2020 Feb 6;11:32. doi: 10.3389/fpls.2020.00032 (PMC7016217; doi:10.3389/fpls.2020.00032)
Supplement: Supplementary file 1 [file DataSheet_1.pdf]

Table S1. Summary of phenotypes and the subcellular localization of eighteen bri1 alleles carrying point mutations.

Subdomain	Mutants	Cellular localization	Phenotype
N-cap	C69Y (bri1-5)	ER	Weak
LRR	G87E (bri1-R1)	PM	Suppress bri1-5
	S196F (bri1_194H8)	PM	unknown
	S157F (bri1_102D4)	PM	unknown
	T750I (bri1-102)	PM	Strong
	S399F (bri1-120)	PM	Weak
	S662F (bri1-9)	ER	Weak
Island domain	G644D (bri1-6)	PM	Intermediate
	G643E (sud1)	PM	Gain of function
	G611E (bri1-113)	PM	Strong
	G613S (bri1-7)	PM	Intermediate
Kinase domain	A909T (bri1-1)	PM	Strong
	E1078K (bri1-101)	PM	Strong
	A1031T (bri1-103)	PM	Strong
	R983Q (bri1-8)	PM	Intermediate
	G1048D (bri1-115)	PM	Strong
	D1139N (bri1-117)	PM	Strong
	G989I (bri1-301)	PM	Weak

Table S2. Hydrophobicity of BRI1 LRRs. The hydrophobicity, hydrophobic residue number and the grand average of hydropathy (GRAVY) of each LRR in both wild type BRI1 and its S to F alleles are shown.

LRR	Residue No.	Conserve Serine	Hydrophobicity	GRAVY	Hydrophobicity (S-F)	GRAVY
1	23	80	17.7	0.77	21.3	0.93
2	23	107	8.5	0.37	12.1	0.53
3	26	130	6.4	0.25	10	0.38
4	25	156	2.4	0.10	6	0.24
5	27	181	16.8	0.62	20.4	0.76
6	20	208	0.8	0.04	4.4	0.22
7	23	230	8.7	0.38	12.3	0.53
8	24	253	-1.8	-0.08	1.8	0.08
9	22	277	6.7	0.30	10.3	0.47
10	25	A	0.6	0.02		
11	24	324	5.5	0.23	9.1	0.38
12	25	348	7.5	0.30	11.1	0.44
13	24	373	-4.2	-0.18	-0.6	-0.03
14	24	398	15.9	0.66	19.5	0.81
15	23	Q	-14.1	-0.61		
16	25	448	16.6	0.66	20.2	0.81
17	24	W	-3.4	-0.14		
18	24	D	4	0.17		
19	24	520	-10.2	-0.42	-5.6(S-L)	-0.23
20	24	544	1.2	0.05	4.8	0.20
21	25	N	6.8	0.27		
22	22	662	4.4	0.20	8	0.36
23	24	G	2.3	0.10		
24	24	710	-1.7	-0.07	1.9	0.08
25	24	734	-0.7	-0.03	2.9	0.12

Table S3. Primers used for site-directed mutagenesis in current work.

Name	Sequence (5'-3')
S157F	GAAGTTTCTTAACGTCTCTTTCAATACACTTGATTTTCCCGG
	CCGGGAAAATCAAGTGTATTGAAAGAGACGTTAAGAAACTTC
S196F	GTCGTTGGTTGGGTTCTCTTCGATGGGTGTGGAGAGTTG
	CAACTCTCCACACCCATCGAAGAGAACCCAACCAACGAC
S399F	GCTAACGTTAGATCTCAGCTTCAACAATTTCTCCGGTCCG
	CGGACCGGAGAAATTGTTGAAGCTGAGATCTAACGTTAGC
S662T	GATGTTTCTGGACATGTATTACAACATGTTGTCTGG
	CCAGACAACATGTTGTAATACATGTCCAGAAACATC
S662W	CGATGATGTTTCTGGACATGTGGTACAACATGTTGTCTGGATAC
	GTATCCAGACAACATGTTGTACCACATGTCCAGAAACATCATCG
F662V	CGATGATGTTTCTGGACATGGTTTACAACATGTTGTCTGG
	CCAGACAACATGTTGTAAACCATGTCCAGAAACATCATCG
F662L	GATGTTTCTGGACATGTTGTACAACATGTTGTCTGG
	CCAGACAACATGTTGTACAACATGTCCAGAAACATC
S662A	GATGATGTTTCTGGACATGGCTTACAACATGTTGTCTG
	CAGACAACATGTTGTAAGCCATGTCCAGAAACATCATC
S662T	GATGATGTTTCTGGACATGACTTACAACATGTTGTCTGG
	CCAGACAACATGTTGTAAGTCATGTCCAGAAACATCATC
S662C	GATGTTTCTGGACATGTGTTACAACATGTTGTCTGG
	CCAGACAACATGTTGTAACACATGTCCAGAAACATC

S80F	GTTACTTCGATTGATCTCTTCTCCAAGCCTCTCAACGTC
	GACGTTGAGAGGCTTGGAGAAGAGATCAATCGAAGTAAC
S107F	GATTAGAGTCTCTGTTTCTCTTTAACTCACACATCAATGGCTCC
	GGAGCCATTGATGTGTGAGTTAAAGAGAAACAGAGACTCTAATC
S156F	GTCTGAAGTTTCTTAACGTCTTTTCCAATACACTTGATTTTCC
	GGAAAATCAAGTGTATTGGAAAAGACGTTAAGAAACTTCAGAC
S181F	CTTGGAAGTTCTGGATCTTTTTGCGAATTCAATCTCCGGTG
	CACCGGAGATTGAATTCGCAAAAAGATCCAGAACTTCCAAG
S208F	GAGTTGAAACATTTAGCGATTTTCGGAAACAAAATCAGTGGAG
	CTCCACTGATTTTGTTTCCGAAAATCGCTAAATGTTTCAACTC
S230F	CTCGAGTTTCTCGATGTTTTCTCCAACAATTTCTCCACTG
	CAGTGGAGAAATTGTTGGAGAAAACATCGAGAAACTCGAG
S253F	CTGCAACATCTTGACATCTTCGGGAACAAATTATCCGG
	CCGGATAATTTGTTCCCGAAGATGTCAAGATGTTGCAG
S277F	CTCAAGTTGTTGAACATCTTTAGTAACCAATTCGTCGGAC
	GTCCGACGAATTGGTTACTAAAGATGTTCAACAACTTGAG
S324F	CACTCACTGGTCTCGATCTCTTTGGAAATCATTTCTACGGTG
	CACCGTAGAAATGATTTCCAAAGAGATCGAGACCAGTGAGTG
S348F	CTCGAATCACTCGCGTTGTTTAGTAACAACTTCTCTGGCG
	CGCCAGAGAAGTTGTTACTAAACAACGCGAGTGATTCGAG
S373F	CTCAAAGTACTTGATCTGTTTTTCAACGAGTTTTCCGGCG
	CGCCGGAAAACTCGTTGAAAAACAGATCAAGTACTTTGAG

S398F	GTTGCTAACGTTAGATCTCTTCTCCAACAATTTCTCCGGTC
	GACCGGAGAAATTGTTGGAGAAGAGATCTAACGTTAGCAAC
S448F	GCTGGTTTCGCTTCACTTGTTCTTCAATTACCTCTCCGGG
	CCCGGAGAGGTAATTGAAGAACAAGTGAAGCGAAACCAGC
S520L	CTTAACTGGATTTCTCTGTTGAATAACCGGTTAACCGGTG
	CACCGGTTAACCGGTTATTCAACAGAGAAATCCAGTTAAG
S544F	CTCGCTATCCTCAAGCTATTCAACAATTCATTCTCCGGG
	CCCGGAGAATGAATTGTTGAATAGCTTGAGGATAGCGAG
S710F	GTTTAAACATTCTTGATCTTTTTAGCAATAAGCTCGATGGGAGG
	CCTCCCATCGAGCTTATTGCTAAAAAGATCAAGAATGTTTAAAC
S734F	GCTTACGGAAATCGATTTGTTTAATAATAATTTGTCTGGTCCG
	CGGACCAGACAAATTATTATTAAACAAATCGATTTCCGTAAGC
S130F	CTTTAACCAGCTTGGATCTATTTAGAAACTCTCTTTCGGGTC
	GACCCGAAAGAGAGTTTCTAAATAGATCCAAGCTGGTTAAAG
S130A	CTTTAACCAGCTTGGATCTAGCTAGAAACTCTCTTTCGGGTC
	GACCCGAAAGAGAGTTTCTAGCTAGATCCAAGCTGGTTAAAG
S130T	CTTTAACCAGCTTGGATCTAACTAGAAACTCTCTTTCGGGTC
	GACCCGAAAGAGAGTTTCTAGTTAGATCCAAGCTGGTTAAAG
S130V	CTTTAACCAGCTTGGATCTAGTTAGAAACTCTCTTTCGGGTC
	GACCCGAAAGAGAGTTTCTAACTAGATCCAAGCTGGTTAAAG
S80T	GTTACTTCGATTGATCTCACCTCCAAGCCTCTCAACGTC
	GACGTTGAGAGGCTTGGAGGTGAGATCAATCGAAGTAAC

S181T	CTTGGAAGTTCTGGATCTTACTGCGAATTCAATCTCCGGTG
	CACCGGAGATTGAATTCGCAGTAAGATCCAGAACTTCCAAG
S107T	GATTAGAGTCTCTGTTTCTCACAAACTCACACATCAATGGCTCC
	GGAGCCATTGATGTGTGAGTTTGTGAGAAACAGAGACTCTAATC
S156T	GTCTGAAGTTTCTTAACGTCACTTCCAATACACTTGATTTTCC
	GGAAAATCAAGTGTATTGGAAGTGACGTTAAGAAACTTCAGAC
S230T	CTCGAGTTTCTCGATGTTACCTCCAACAATTTCTCCACTG
	CAGTGGAGAAATTGTTGGAGGTAACATCGAGAAACTCGAG
S253T	CTGCAACATCTTGACATCACCGGGAACAAATTATCCGG
	CCGGATAATTTGTTCCCGGTGATGTCAAGATGTTGCAG
S398T	GTTGCTAACGTTAGATCTCACCTCCAACAATTTCTCCGGTC
	GACCGGAGAAATTGTTGGAGGTGAGATCTAACGTTAGCAAC
S710T	GTTTAAACATTCTTGATCTTACAAGCAATAAGCTCGATGGGAGG
	CCTCCCATCGAGCTTATTGCTTGTAAGATCAAGAATGTTTAAAC
S135F	CTATCTAGAAACTCTCTTTTCGGTCCTGTAACGACTCTAAC
	GTTAGAGTCGTTACAGGACCGAAAAGAGAGTTTCTAGATAG
S186F	CTTTCTGCGAATTCAATCTTCGGTGCTAACGTCGTTGG
	CCAACGACGTTAGCACCGAAGATTGAATTCGCAGAAAG
S235F	GTTTCCTCCAACAATTTCTTCACTGGGATTCCTTTCCTC
	GAGGAAAGGAATCCCAGTGAAGAAATTGTTGGAGGAAAC
S258F	CTCCGGGAACAAATTATTCGGCGATTTCTCCCGTGC
	GCACGGGAGAAATCGCCGAATAATTTGTTCCCGGAG

S403F	CTCAGCTCCAACAATTTCTTCGGTCCGATTCTCCCAAATC
	GATTTGGGAGAATCGGACCGAAGAAATTGTTGGAGCTGAG
S667F	GTCTTACAACATGTTGTTTGGATACATACCGAAGG
	CCTTCGGTATGTATCCAAACAACATGTTGTAAGAC
S80A	GTTACTTCGATTGATCTCGCCTCCAAGCCTCTCAACGTC
	GACGTTGAGAGGCTTGGAGGCGAGATCAATCGAAGTAAC
S107A	GATTAGAGTCTCTGTTTCTCGCAAACTCACACATCAATGGCTCC
	GGAGCCATTGATGTGTGAGTTTGCGAGAAACAGAGACTCTAATC
Q424S	CTGCAGGAGCTTTACCTTTCGAACAATGGCTTCACCGG
	CCGGTGAAGCCATTGTTCGAAAGGTAAAGCTCCTGCAG
W472S	CTTCGAGATCTGAAACTATCGCTGAATATGTTAGAAGGAG
	CTCCTTCTAACATATTCAGCGATAGTTTCAGATCTCGAAG
D496S	CTTAGAGACTCTGATCCTCAGCTTCAACGATTTAACCGG
	CCGGTTAAATCGTTGAAGCTGAGGATCAGAGTCTCTAAG
N568S	CTTAATCTGGCTTGATCTCAGCACCAATCTCTTCAATGGAAC
	GTTCCATTGAAGAGATTGGTGCTGAGATCAAGCCAGATTAAG

Table S4. cDNA sequences blast from 9 representative plant genomes using Arabidopsis BRI1 protein sequence as a query.

Organism (Version)	Abbreviation	cDNA Sequences
Picea abies (v1.0)	Picab	MA_170g0010
		MA_57173g0030
		MA_177039g0010
		MA_1913g0010
		MA_37224g0010
		MA_103858g0010
		MA_101553g0010
		MA_10427804g0010
		MA_18697g0010
		MA_64117g0010
Arabidopsis thaliana (TAIR10)	Arath	AT1G17230
		AT1G28440
		AT1G55610
		AT1G74360
		AT2G01950
		AT2G02220
		AT2G33170
		AT3G13380
		AT3G24240
		AT3G49670
		AT4G20140
		AT4G20270
		AT4G28650
		AT4G39400
		AT5G07280
		AT5G44700
		AT5G48940
		AT5G53890
		AT5G63930
		AT5G65700
Brachypodium distachyon (v3.1)	Bradi	Bradi1g07180
		Bradi1g26900
		Bradi1g33480
		Bradi1g37190
		Bradi1g46450
		Bradi1g57900
		Bradi1g58460
		Bradi1g69097

		Bradi1g72572
		Bradi2g48279
		Bradi2g58780
		Bradi3g04176
		Bradi3g04187
		Bradi3g04217
		Bradi3g04227
		Bradi3g06980
		Bradi3g21400
		Bradi3g49370
		Bradi4g27440
		Bradi5g25790
Brassica rapa (v1.3)	Brara	Brara.A00065
		Brara.A00859
		Brara.A01093
		Brara.B01401
		Brara.B03093
		Brara.D02007
		Brara.E02779
		Brara.F01212
		Brara.F02442
		Brara.F03427
		Brara.F03441
		Brara.F03851
		Brara.G00535
		Brara.G03116
		Brara.G03184
		Brara.H00042
		Brara.H01801
		Brara.J00880
		Brara.J02502
		Brara.K01799
Medicago truncatula (Mt4.0v1)	Medtr	Medtr1g047670
		Medtr1g079520
		Medtr1g080440
		Medtr1g100787
		Medtr1g102500
		Medtr2g014560
		Medtr2g070020
		Medtr2g078810
		Medtr3g009400
		Medtr3g095100
		Medtr3g437630

		Medtr3g449390
		Medtr4g036575
		Medtr4g088320
		Medtr4g094790
		Medtr4g109170
		Medtr5g014700
		Medtr5g045910
		Medtr7g059285
		Medtr8g023720
Oryza sativa (v7_JGI)	Orisa	LOC_Os01g52050
		LOC_Os01g68870
		LOC_Os02g02140
		LOC_Os02g02490
		LOC_Os02g05910
		LOC_Os02g05930
		LOC_Os02g05940
		LOC_Os02g05960
		LOC_Os02g05970
		LOC_Os02g05980
		LOC_Os02g41890
		LOC_Os04g57630
		LOC_Os06g47700
		LOC_Os06g47720
		LOC_Os06g47740
		LOC_Os07g04190
		LOC_Os07g05740
		LOC_Os08g25380
		LOC_Os09g12240
		LOC_Os10g02500
Amborella trichopoda (v1.0)	Ambtr	evm_27.TU.AmTr_v1.0_scaffold00008.36
		evm_27.TU.AmTr_v1.0_scaffold00009.24
		evm_27.TU.AmTr_v1.0_scaffold00019.207
		evm_27.TU.AmTr_v1.0_scaffold00021.186
		evm_27.TU.AmTr_v1.0_scaffold00024.267
		evm_27.TU.AmTr_v1.0_scaffold00024.56
		evm_27.TU.AmTr_v1.0_scaffold00029.177
		evm_27.TU.AmTr_v1.0_scaffold00032.242
		evm_27.TU.AmTr_v1.0_scaffold00033.36
		evm_27.TU.AmTr_v1.0_scaffold00039.8
		evm_27.TU.AmTr_v1.0_scaffold00055.1
		evm_27.TU.AmTr_v1.0_scaffold00057.129
		evm_27.TU.AmTr_v1.0_scaffold00069.214
		evm_27.TU.AmTr_v1.0_scaffold00071.66

		evm_27.TU.AmTr_v1.0_scaffold00078.169
		evm_27.TU.AmTr_v1.0_scaffold00087.11
		evm_27.TU.AmTr_v1.0_scaffold00092.78
		evm_27.TU.AmTr_v1.0_scaffold00102.64
		evm_27.TU.AmTr_v1.0_scaffold00120.45
		evm_27.TU.AmTr_v1.0_scaffold00160.2
Arabidopsis lyrata (v2.1)	Araly	AL1G29470
		AL1G42070
		AL1G63680
		AL2G31980
		AL2G34210
		AL3G25230
		AL3G38940
		AL4G18950
		AL4G28730
		AL5G11090
		AL5G11490
		AL5G29520
		AL6G17520
		AL7G11420
		AL7G23890
		AL7G33680
		AL8G22150
		AL8G28960
		AL8G41170
		AL8G43410
Solanum lycopersicum (iTAG2.4)	Solly	Solyc01g080770.2
		Solyc01g102870.1
		Solyc02g077630.2
		Solyc03g006300.1
		Solyc03g007050.2
		Solyc03g026040.2
		Solyc03g112580.2
		Solyc03g115610.2
		Solyc04g051510.1
		Solyc04g064940.2
		Solyc05g007230.2
		Solyc05g051640.2
		Solyc06g069650.2
		Solyc07g056410.2
		Solyc07g066230.2
		Solyc08g061560.2
		Solyc09g061940.1

		Solyc09g091260.2
		Solyc09g098420.1
		Solyc12g098100.1
