# Supplementary material for: The Evolutionarily Conserved Serine Residues in BRI1 LRR Motifs Are Critical for Protein Secretion
Source: Front Plant Sci. 2020 Feb 6;11:32. doi: 10.3389/fpls.2020.00032 (PMC7016217; doi:10.3389/fpls.2020.00032)
Supplement: Supplementary file 2 [file DataSheet_2.pdf]

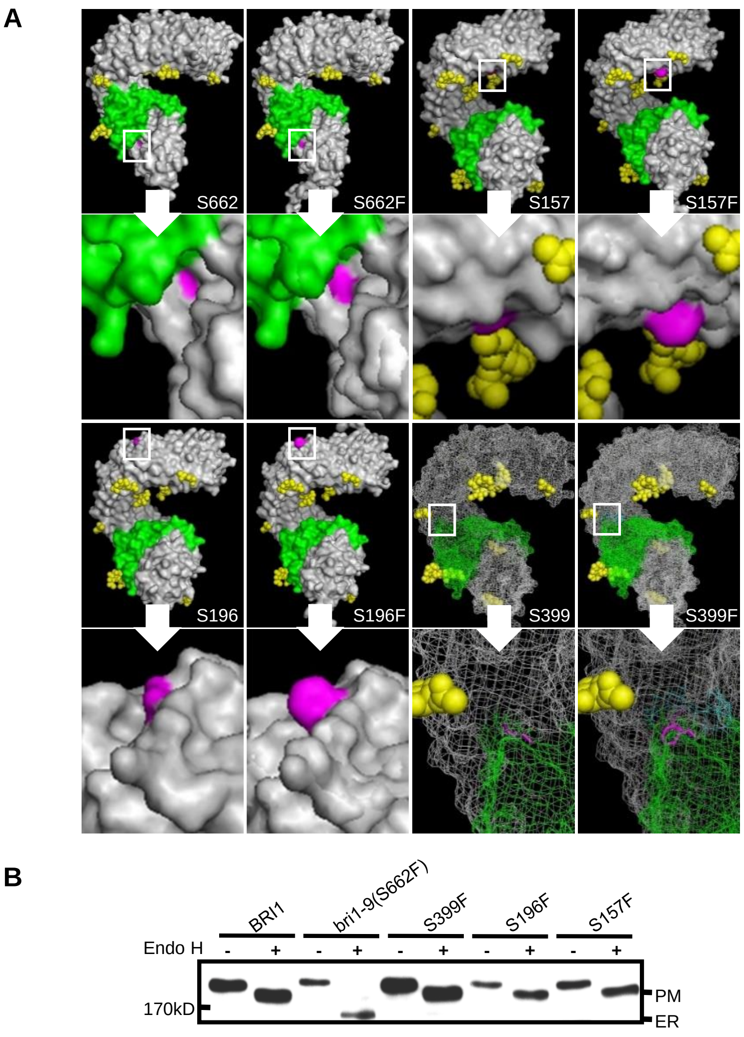

SUPPLEMENTARY FIGURE 1. Subcellular localization of BRI1 variants carrying a random Ser to Phe mutation at the LRR domain.

(A) Surface exposures modeling of known Ser to Phe mutations. Based on BRI1 (PDB 3RGX), the surface model and mesh model were displayed, the solvent-accessible surface of the protein were shown with PyMol software. Island domain and mutation sites were highlighted in green and magenta, respectively. The N-glycans were indicated by yellow spheres. The surface exposures of the mutation sites were enlarged and shown below. **(B)** Western blot analyses of wild-type (WT) and mutated BRI1-GFPs. Total proteins from WT seedlings expressing *BRI1-GFP* and *bri1-GFPs* holding single S to F substitution in the LRR domain were treated with or without Endo H to differentiate the protein mobility shift. The bands corresponding to BRI1-GFP at plasma membrane (PM) and the endoplasmic reticulum (ER) were marked.

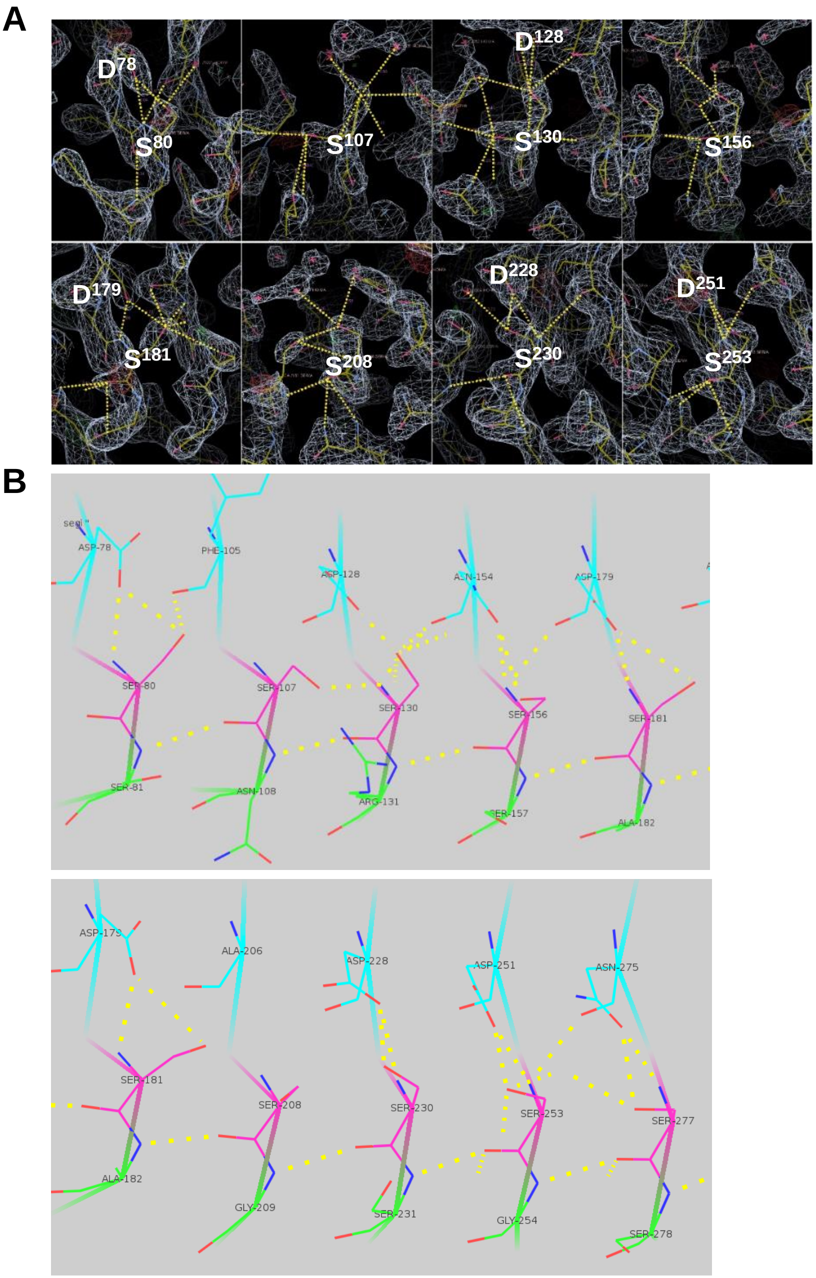

SUPPLEMENTARY FIGURE 2. The modeling of polar contacts among Ser* residues from BRI1 LRR1 to LRR8.
(A) The polar contacts of a single Ser* residue with adjacent residues within 3.5 Å restriction shown in WinCoot (0.8.6.1). **(B)** The continuous polar contacts of Ser* residue from LRR1 to LRR8 within 3.5 Å restriction shown in WinCoot (0.8.6.1)

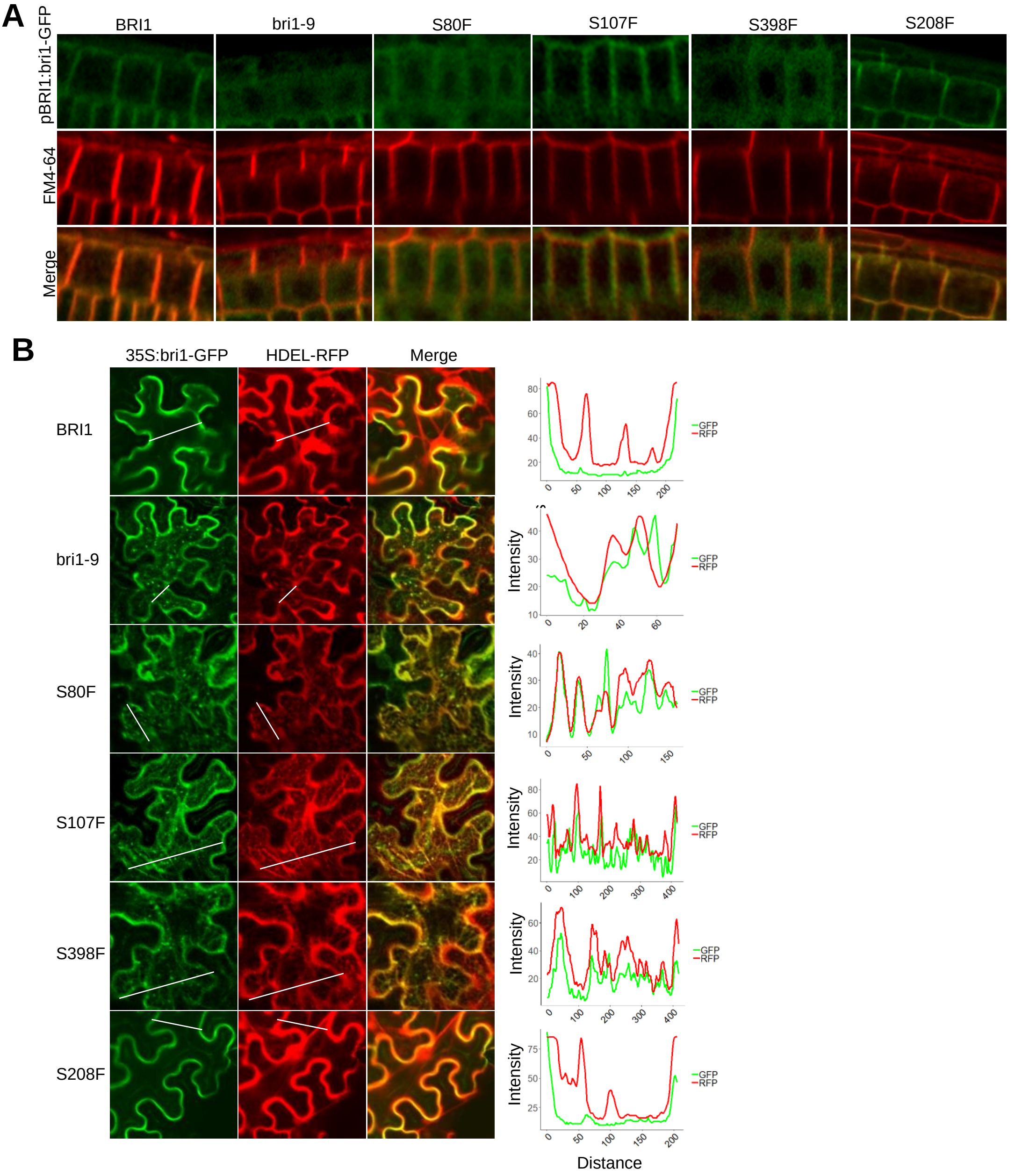

SUPPLEMENTARY FIGURE 3. Confocal microscopic analyses of BRI1-GFP variants with different secretion patterns.

(A) Confocal analysis of subcellular localization of *bri1-GFPs* in root tips of 6-day-old light-grown *bri1-301* seedlings expressing *pBRI1:bri1-GFPs*. Seedlings were stained with FM4-64 (Invitrogen) for 2 min under a concentration of 10 μM to denote the PM, and unbound FM4-64 was washed out. **(B)** Three bri1-GPF variants showing different ER retention pattern were confirmed by transient expression in *Agrobacterium*-infiltrated tobacco leaves. BRI1-GFP and bri1-9-GFP were used as PM and ER-localized proteins, respectively. HEDL-RFP used as ER-localization marker. The fluorescent intensity of the noted line were analyzed in Image J.

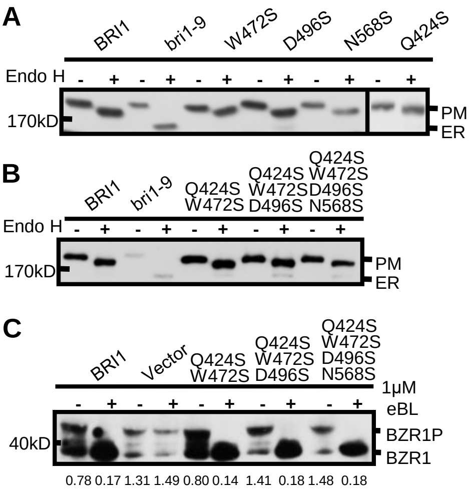

SUPPLEMENTARY FIGURE 4. Western blot analysis of BRI1 variants carrying mutations on the variable residues along the S*-chain.

(A) and **(B)** Endo H assay of bri1 variants with substitutions at non-serine residues from LRR15-LRR20. Total protein were extracted from 5 T1 *bri1-301* seedlings expressing *BRI1-GFP* and *bri1-GFPs,* and detected with anti-GFP antibody. BRI1-GFP and bri1-9-GFP were used as PM and ER-localized indicator protein, respectively. **(C)** The dephosphorylation of BZR1. The T2 *bri1-301* lines expressing similar level of various *bri1-GFPs* **(Fig. 5D)** were incubated in liquid half MS medium supplemented with or without 1.0 μM eBL for 1 h. Total protein were extracted, separated in gel and detected with anti-BZR1 antibody. The ratio of the gray value between the phosphorylated and dephosphorylated BZR1 bands were analyzed in ImageJ.

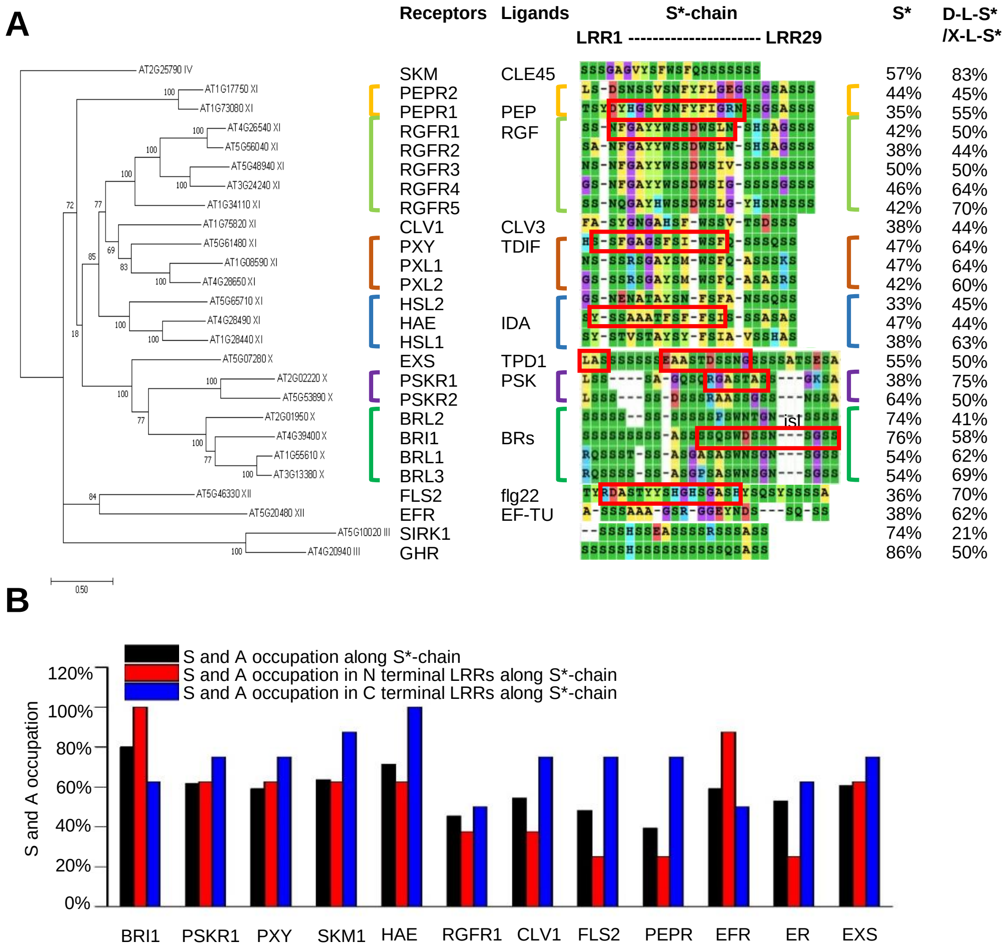

SUPPLEMENTARY FIGURE 5. The appearance of Ser* residues in the known Arabidopsis LRR-RLKs.

(A) The phylogenic analyses of some known Arabidopsis LRR-RLKs and their close homologs. The analyses were based upon the full coding sequences. The bar represented a mutation rate of 0.50 per site. Bootstrap values were shown near the nodes. The presence of Ser* residue as well as the ratio of D-L-S*/X-L-S* (X indicated variable residues) were also analyzed. Island domain was indicated as “isl”. LRRs reported to be involved in ligand binding were highlighted in red boxes. **(B)** The occupancy of Ser and Ala residues. The presences of Ser or Ala along the S*-chain as well as those lying in the N or C terminals were shown as percentage.

MATERIALS AND METHODS

Transformation of Tobacco Leaves and Confocal Microscopy

Leaves from 6-week-old tobacco plants (*Nicotiana benthamiana*) were used for transient expression of *p35S:bri1-GFPs* and *p35S:HDEL-RFP* via *Agrobacterium*-mediated infiltration (Sparkes et al. 2006). After 36 hours for infiltration, the fluorescence was observed using confocal microscope. Tobacco leaves expressing *p35S:bri1-GFPs* and *p35S:HDEL-RFP* or root tips expressing *pBRI1: bri1-GFPs* were observed using Olympus FluoView™ FV1000 Olympus confocal microscope and visualized with FV10-ASW 1.7 Viewer. GFP and RFP were excited using 488 and 543 nm laser light, respectively. Images were acquired with a 0.50 mm Z step at a resolution of 1024 × 1024 pixel using a 20× objective.

REFERENCES

Sparkes, I.A., Runions, J., Kearns, A. and Hawes, C. (2006). Rapid, transient expression of fluorescent fusion proteins in tobacco plants and generation of stably transformed plants. *Nat. Protoc.* 1, 2019-2025. Doi: 10.1038/nprot.2006.286
